# Method for the validation of immunohistochemical staining using SCID mouse xenografts: Expression of CD40 and CD154 in human non-small cell lung cancer

**DOI:** 10.3892/or.2013.2275

**Published:** 2013-02-05

**Authors:** KEIDAI ISHIKAWA, MASAKI MIYAMOTO, TATSUYA YOSHIOKA, MASATOSHI KADOYA, LI LI, ROSHAN MISHRA, KAZUOMI ICHINOKAWA, YASUHITO SHOJI, YOSHIYUKI MATSUMURA, YASUHIRO HIDA, KICHIZO KAGA, TATSUYA KATO, MITSUHITO KAJI, TOSHIRO OHBUCHI, TOMOO ITOH, HIROTOSHI DOSAKA-AKITA, YOSHIRO MATSUI, SATOSHI HIRANO

**Affiliations:** 1 Department of Gastroenterological Surgery II, Hokkaido University Graduate School of Medicine, Sapporo, Hokkaido 060-8638; 2 Department of Cardiovascular and Thoracic Surgery, Hokkaido University Graduate School of Medicine, Sapporo, Hokkaido 060-8638; 3 Department of Thoracic Surgery, Sapporo-Minamisanjo Hospital, Sapporo, Hokkaido 060-0063; 4 Department of Thoracic Surgery, Minami-ichijo Hospital, Sapporo, Hokkaido 060-0061; 5 Department of Diagnostic Pathology, Kobe University Hospital, Kobe, Hyogo 650-0017; 6 Department of Medical Oncology, Division of Cancer Medicine, Hokkaido University Graduate School of Medicine, Sapporo, Hokkaido 060-8638, Japan

**Keywords:** CD40, CD154, lung cancer, immunohistochemistry, positive control, xenograft

## Abstract

This report proposes a concept for the standardization of immunohistochemical evaluation. Immunohistochemical staining has several problems associated with the sensitivity of the technical process and standardization of the assessment of potent staining. We provided data focusing on this concept through immunostaining for CD154 in non-small cell lung cancer (NSCLC). We used two types of anti-CD154 antibody as primary antibodies in immunohistochemical staining, as previously reported. Western blot analysis confirmed strong CD154 expression in the cultured cell line PC10, but not in LK2. We also assessed CD154 expression in SCID mouse xenografts of these cell lines. SCID xenograft data on western blot analysis were consistent with those of cultured cell lines. These xenografts could thus be used as positive or negative tissue controls for CD154 immunostaining. Primary antibodies should therefore be confirmed as recognizing target lesions, while control tissue specimens should be objectively confirmed as having target products using another experimental method. Our method would allow results to be unified at more than one laboratory and could act as an objective control assessment method in immunohistochemistry.

## Introduction

Immunohistochemical staining is associated with several problems related to the sensitivity of the technical process and standardization of the assessment of potent staining, including positive control tissue samples. Previous reports have described ambiguous positive or negative controls when immunohistochemical staining was performed, particularly when no internal positive controls were used. One of the reasons for this ambiguity is that the control tissue specimens themselves were not objectively confirmed as having target products by another experimental method.

CD40 is a 42–48-kDa transmembrane glycoprotein belonging to the tumor necrosis factor (TNF) receptor superfamily (1,2). Its presence was initially described on B cells and in bladder carcinoma (3), but it is also reportedly expressed on monocytes (4), dendritic cells (5), fibroblasts (6), tonsils (7), thymic epithelial cells (8) and endothelial cells (9).

The ligand of CD40 (CD40L, CD154), a 39-kDa membrane glycoprotein, is expressed on T cells, basophils and mast cells (10,11). Interaction between CD40 with CD154 induces proliferation, germinal center formation and allows for the generation of B cells that secrete IgE following isotype switching (12–15). Recent reports have demonstrated CD154 expression in breast cancer (16), thyroid cancer (17) and coronary diseases (18). However, CD154 expression in lung cancer has not been widely studied (19). Consequently, we performed immunohistochemistry for CD40 and CD154 in 129 non-small cell lung cancer (NSCLC) patient tissue samples (20). In the present study, we propose an approach for standardizing the evaluation of control specimens of immunohistochemistry.

## Materials and methods

### Cell lines

Human lung cancer cell lines were obtained from the Japanese Cancer Research Resources Bank (Tokyo, Japan). PC10, LC-1 and LK2 were grown in RPMI-1640 (Sigma-Aldrich Co., Ltd., Irvine, CA, USA) with 10% fetal bovine serum (FBS), and 1% penicillin/streptomycin (p/s). ABC-1 was maintained in minimum essential medium Eagle (M-EME; Sigma-Aldrich Co., Ltd.,) with 10% FBS and 1% p/s. All cell lines were maintained in a humidified incubator with 5% CO_2_ in air at 37°C.

### Mice and tumor xenograft models

CB17/SCID mice were obtained from Charles River Japan (Yokohama, Japan). All mice were female, 4–6 weeks of age, and were maintained under specific pathogen-free conditions. All animal procedures were in accordance with the guidelines of the Hokkaido University Institutional Animal Care and Use Committee using an approved protocol. ABC-1, LC-1, LK2 and PC10 cells (5×10^6^) were subcutaneously injected in a volume of 100 μl of phosphate-buffered saline into the left flank region of each CB17/SCID mouse. When tumor diameter exceeded 10 mm, mice were sacrificed and tumors were separated to 2 blocks: one block was frozen using liquid nitrogen to extract proteins for western blot analysis, and the other was immersed in formalin for immunohistological analysis.

### Reagents and antibodies

Anti-CD154 rabbit polyclonal antibody (C-20:sc-978) and anti-CD40 rabbit polyclonal antibody (C-20:sc-975) were purchased from Santa Cruz Biotechnology (Santa Cruz, CA, USA), and anti-CD154 mouse monoclonal antibody (TRAP1:IM1842) was purchased from Immunotech (Marseille Cedex, France). Anti-CD40 mouse monoclonal antibody (11E9) was purchased from Novocastra (Newcastle, UK).

Peroxidase-conjugated goat F(ab’)2 anti-rabbit IgG and peroxidase-conjugated goat F(ab’)2 anti-mouse IgG were purchased from Jackson ImmunoResearch (West Grove, PA, USA). Negative control rabbit immunoglobulin fraction (normal) (X0903), negative control mouse IgG1 (X0941) and negative control mouse IgG2b (X0944) were purchased from Dako Japan (Kyoto, Japan). Recombinant human soluble CD40 ligand protein (TRAP1:gp39) was purchased from Chemicon International Inc. (Billerica, MA, USA).

### Western blot analysis

Western blot analysis was performed in order to analyze CD154 expression in lung cancer cells. Lysates from cell lines and lysates from SCID mouse xenografts were prepared in SDS buffer containing 62.5 mm Tris-HCl (pH 6.8), 2% w/v SDS, 10% glycerol, 50 mm DTT, 0.1% w/v bromphenol blue and 1 mm PMSF. Total proteins (20 μg) were electrophoresed in 15% SDS-polyacrylamyde gels and were transferred onto nitrocellulose membranes.

Anti-CD154 rabbit polyclonal antibody (C-20:sc-978, 1:100) and anti-CD154 mouse monoclonal antibody (TRAP1:IM1842, 1:20) were used as primary antibodies for CD154 and anti-CD40 rabbit polyclonal antibody (C-20:sc-975, 1:200) was used as the primary antibody for CD40. The appropriate peroxidase-conjugated goat anti-rabbit or anti-mouse IgG was used as the secondary antibody (1:10,000).

Detection of bound antibodies was performed using the ECL system (Amersham, Aylesbury, UK). The recombinant human soluble CD40 ligand (rhsCD40L, rhsCD154) (Chemicon International) was used as a positive control for CD154. Lysates from normal human peripheral blood mononuclear cells (PBMCs) were used as a positive control for CD40.

### Patients and tissue specimens

Whole surgical specimens of resected NSCLC were utilized in this study. Patients enrolled in the study showed no signs of metastases to secondary sites and had received no prior anticancer treatments. Cases of in-hospital and non-cancer-related deaths were excluded. We examined 129 NSCLC surgical specimens meeting these criteria from patients undergoing curative resection of the primary tumor, including systematic lymph node dissection. Resected specimens were examined histopathologically after staining with hematoxylin and eosin. A single section, from deep in the tumor specimen, was selected for analysis, and at least two-independent pathologists performed each diagnosis.

### Immunohistochemistry

Immunohistochemical reactions were carried out using the universal immuno-enzyme polymer method. Tumors from SCID mouse xenograft models and surgical specimens were fixed in 10% formalin solution and embedded in paraffin for sectioning at 4 μm. Sections were then deparaffinized in xylene, dehydrated through a graded ethanol series, and were either left untreated or treated with a pressure cooker for 2 min.

Endogenous peroxidase activity was blocked by a 10-min incubation with hydrogen peroxide. Following three washes in phosphate-buffered saline with 1% Tween-20 (PBS-T), sections were incubated in 10% normal goat serum (Histofine SAB-PO kit; Nichirei, Tokyo, Japan) for 30 min.

Samples were then incubated overnight with an anti-CD154 rabbit polyclonal antibody (1:500 dilution, C-20:sc-978) at 4°C. In addition, serial tissue sections of each sample were separately incubated overnight with an anti-CD154 mouse monoclonal antibody (1:25 dilution, TRAP1:IM1842), and with an anti-CD40 mouse monoclonal antibody (1:40) at 4°C. Isotype-matched negative control mouse IgG1 (X0931), negative control rabbit immunoglobulin fraction (X0903) and negative control mouse IgG2b (X0944) were used as primary antibodies. A mixture of C-20:sc-978 or TRAP1:IM1842 antibody with 0.1 mg/ml recombinant human soluble CD154 protein (rhsCD154) boiled for 2 min and incubated for 5 or 90 min at room temperature was also used as a primary antibody. Following three additional washes, sections were incubated for 30 min at room temperature with Histofine MAX-PO (Multi) (Histofine SAB-PO kit; Nichirei).

Reaction products were visualized by incubation for ~5 min with 3,3′-diaminobenzidine tetrahydrochloride (Nichirei), followed by washing in distilled water. Sections were counterstained in hematoxylin for 1 min, and then mounted in Permount (micro slides; Muto-Glass, Tokyo, Japan). Immunostained sections were evaluated under a microscope (Olympus Optical Co., Ltd., Tokyo, Japan).

## Results

### Expression of CD154 in non-small lung cancer cell lines in vitro and SCID xenografts in vivo

**Figure 1:**
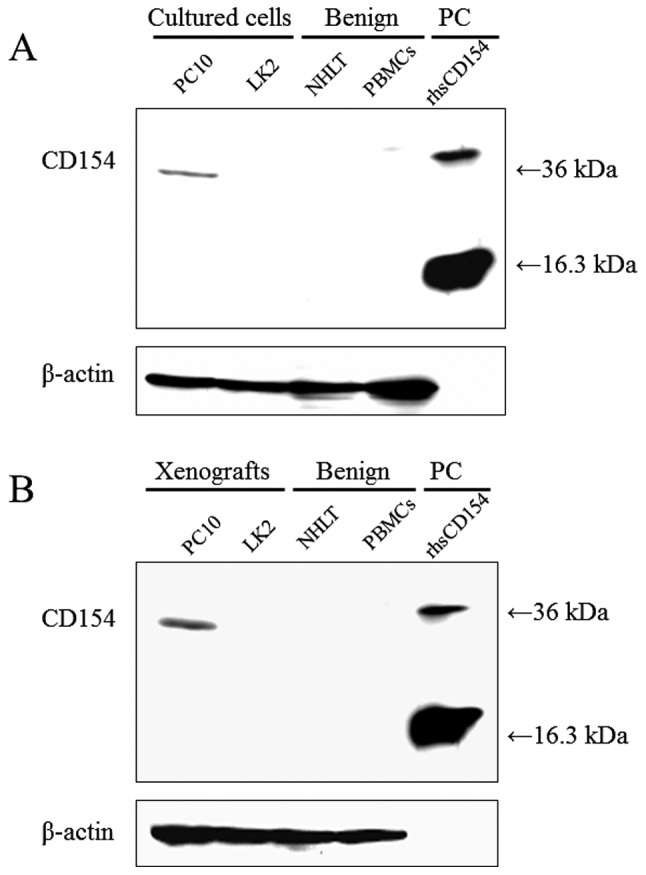
Western blotting for CD154. (A) Lysates from human lung cancer cell lines and homogenized NHLT were subjected to western blotting. Recombinant human soluble CD154 protein (rhsCD154) was used as a PC. CD154 was strongly detected at 36 kDa in lysates from PC10 cells. (B) Western blotting of CD154, lysates from CB17/SCID mouse xenograft models. Lysates from implanted tumors derived from human lung cancer cell lines and NHLT were subjected to western blotting. Each lung cell line was implanted subcutaneously into the left flanks of CB17/SCID mice. NHLT, normal human lung tissue; PBMCs, peripheral blood mononuclear cells. PC, positive control.

Using western blot analysis, with C-20:sc-978 as a primary antibody, CD154 expression was detected at 36 kDa in the lane loaded with the cultured cell line PC10, but was not detected with LK2, homogenized normal human lung tissue (NHLT) or peripheral blood mononuclear cells (PBMCs) (Fig. 1A). CD154 expression in lysates from SCID mouse xenografts was very similar to that in lysates from cultured cell lines (Fig. 1B). Conversely, using TRAP1:IM1842 as a primary antibody, no bands were detected, even in the lane loaded with rhsCD154 (data not shown).

### Immunohistochemical staining for CD154 in SCID mouse xenograft models

**Figure 2:**
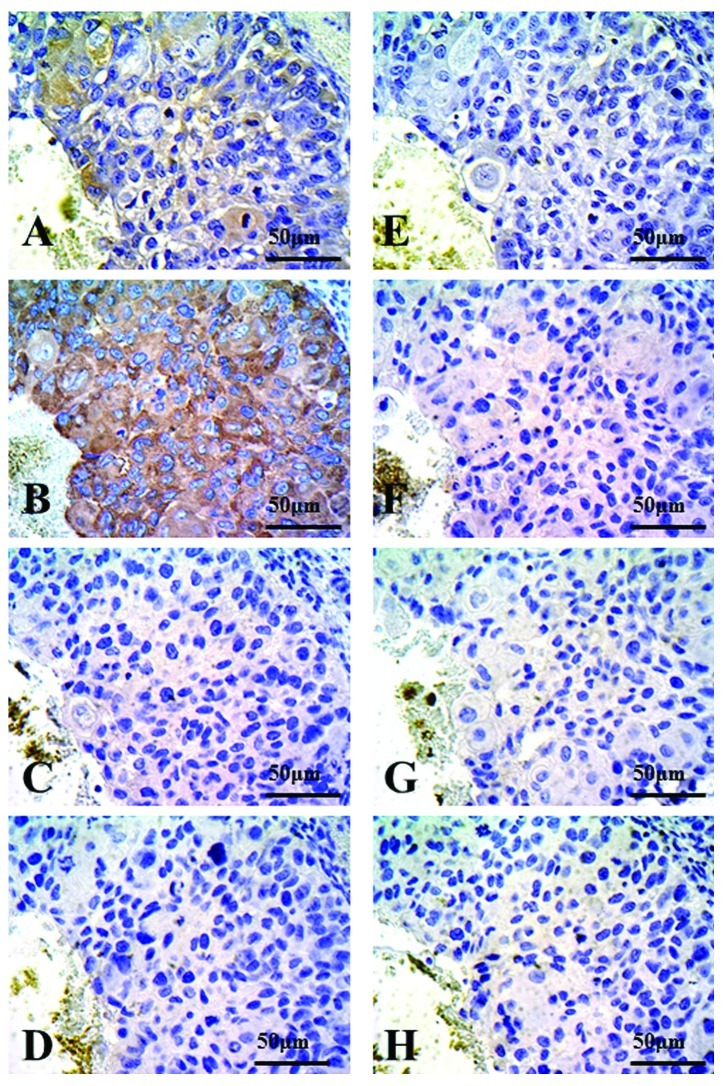
Immunohistochemical staining for CD154, PC10 xenograft models. Using western blot analysis, PC10 xenografts exhibited CD154-positive cells. Autoclaved or non-autoclaved refers to the presence of heat treatment using a pressure cooker. (A) Anti-CD154 antibody (C-20:sc-978, 1:500 dilution), non-autoclaved. (B) Anti-CD154 antibody (C-20:sc-978, 1:500 dilution), autoclaved. (C) Anti-CD154 antibody (C-20:sc-978, 1:500 dilution), autoclaved, with ^*^rhsCD154. (D) Rabbit control IgG. (E) Anti-CD154 antibody (TRAP1:IM1842, 1:25 dilution), non-autoclaved. (F) Anti-CD154 antibody (TRAP1:IM1842, 1:25 dilution), autoclaved. (G) Anti-CD154 antibody (TRAP1:IM1842, 1:25 dilution), autoclaved, with ^*^rhsCD154. (H) Mouse control IgG. ^*^rhsCD154, recombinant human soluble CD154 (CD40 ligand).

**Figure 3:**
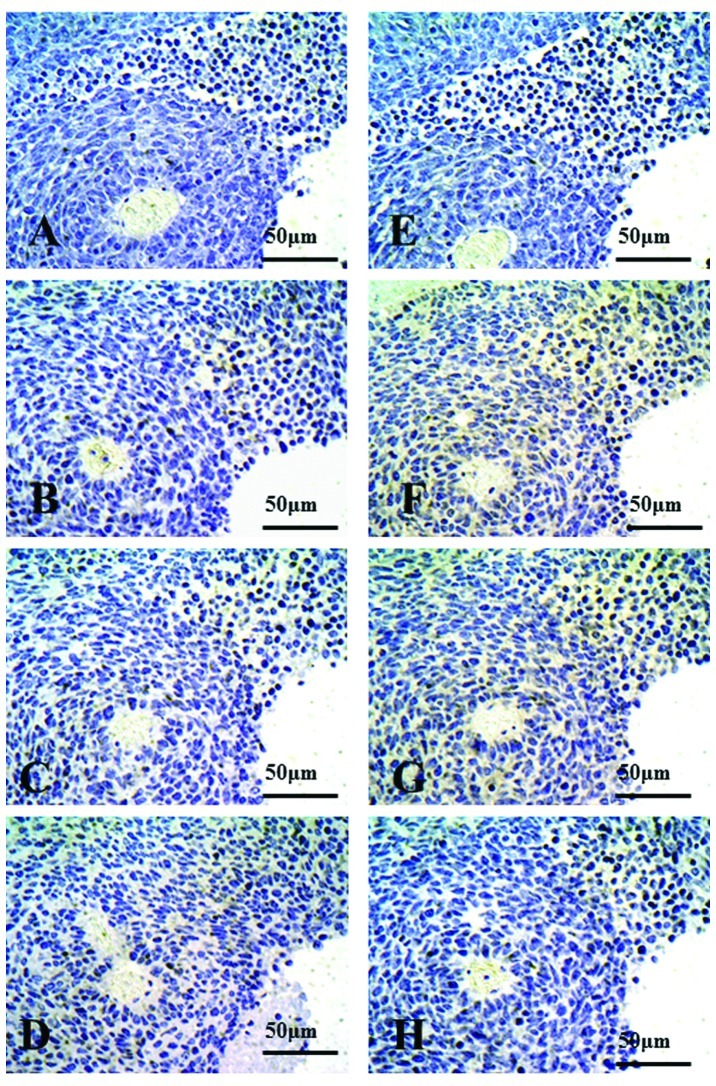
Immunohistochemical staining for CD154, LK2 xenograft models. Using western blot analysis, LK2 xenografts exhibited CD154-negative cells. Autoclaved or non-autoclaved refers to the presence of heat treatment using a pressure cooker. (A) Anti-CD154 antibody (C-20:sc-978, 1:500 dilution), non-autoclaved. (B) Anti-CD154 antibody (C-20:sc-978, 1:500 dilution), autoclaved. (C) Anti-CD154 antibody (C-20:sc-978, 1:500 dilution), autoclaved, with ^*^rhsCD154. (D) Rabbit control IgG. (E) Anti-CD154 antibody (TRAP1:IM1842, 1:25 dilution), non-autoclaved. (F) Anti-CD154 antibody (TRAP1:IM1842, 1:25 dilution), autoclaved. (G) Anti-CD154 antibody (TRAP1:IM1842, 1:25 dilution), autoclaved, with ^*^rhsCD154. (H) Mouse control IgG. ^*^rhsCD154, recombinant human soluble CD154 (CD40 ligand).

SCID mouse xenograft models using two cell lines, PC10 and LK2, were established. When C-20:sc-978 was used as a primary antibody, CD154 expression in the PC10 xenograft was weakly detected without pressure cooker treatment (Fig. 2A). By contrast, CD154 expression was strongly detected in the cytoplasm and cell membranes in the PC10 xenograft with pressure cooker treatment (Fig. 2B). When rhsCD154 was added to C-20:sc-978 and incubated for 90 min at room temperature, CD154 staining in the xenograft PC10 was markedly decreased (Fig. 2C). On the other hand, CD154 expression was not detected in the LK2 xenograft, with (Fig. 3B) or without (Fig. 3A) pressure cooker treatment. When rhsCD154 was added to C-20:sc-978 for 90 min, no expression of CD154 in the LK2 xenograft was detected (Fig. 3C).

When TRAP1:IM1842 was used as a primary antibody, slight expression of CD154 in the PC10 xenograft was seen without pressure cooker treatment (Fig. 2E). CD154 expression was weakly detected in the cytoplasm in the PC10 xenograft with pressure cooker treatment (Fig. 2F). After adding rhsCD154 to TRAP1:IM1842 for 90 min, slight expression of CD154 in the PC10 xenograft was detected (Fig. 2G).

Expression of CD154 in the LK2 xenograft was scarcely seen under all conditions; without pressure cooker treatment (Fig. 3E), with pressure cooker treatment (Fig. 3F) or after adding rhsCD154 to TRAP1:IM1842 for 90 min (Fig. 3G). In addition, after adding rhCD154 to C-20:sc978 or TRAP1:IM1842 for only 5 min, no difference was seen in CD154 staining (data not shown). There were no CD154-positive cells seen in xenografts when control IgG was used as a primary antibody (Figs. 2D and H, and 3D and H).

### Immunohistochemical staining for CD154 in human lung tissues

**Figure 4:**
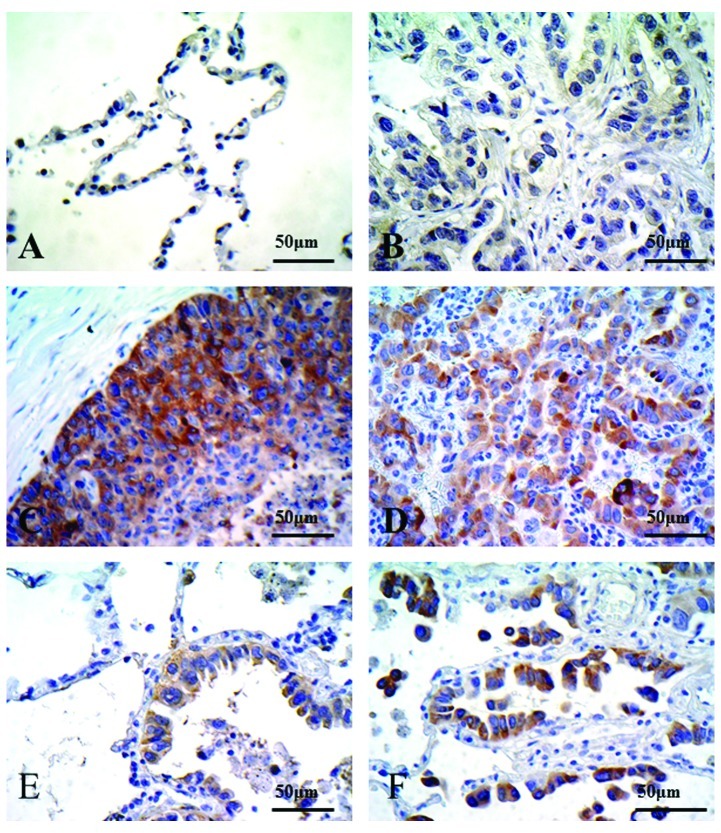
Immunohistochemical staining for CD154 (CD40 ligand) in human lung tissues. (A) Normal alveolar cells without staining for CD154 expression. (B) Adenocarcinoma without staining for CD154. (C) Squamous cell carcinoma with strong staining for CD154 expression. (D) Adenocarcinoma with strong staining in the cytoplasm and cell membrane for CD154. (E) Bronchioloalveolar carcinoma with strong staining for CD154 at edge of foci. (F) Bronchioloalveolar carcinoma with strong staining for CD154 at edge of foci.

Preliminary immunohistochemical staining of lung tissues from 30 patients was performed using C-20:sc-978 as a primary antibody. We used the PC10 xenograft as the positive control for CD154, and the LK2 xenograft as the negative control. Representative photomicrographs are shown in Fig. 3. No CD154 staining was observed in any of the normal alveolar cells (Fig. 4A). Although some adenocarcinoma cases showed no CD154 staining (Fig. 4B), CD154 staining was observed in squamous cell carcinoma (Fig. 4C), adenocarcinoma (Fig. 4D) and bronchioloalveolar carcinomas (BACs) (Fig. 4E and F). Immunostaining patterns in CD154-positive cases in human lung tissue were very similar to the PC10 SCID xenograft, with strong staining in the cytoplasm, particularly in the cell membranes. Thus, there were numerous differences in staining intensity in lung tissues.

### Consistency of CD40 expression results between western blotting and immunostaining

**Figure 5:**
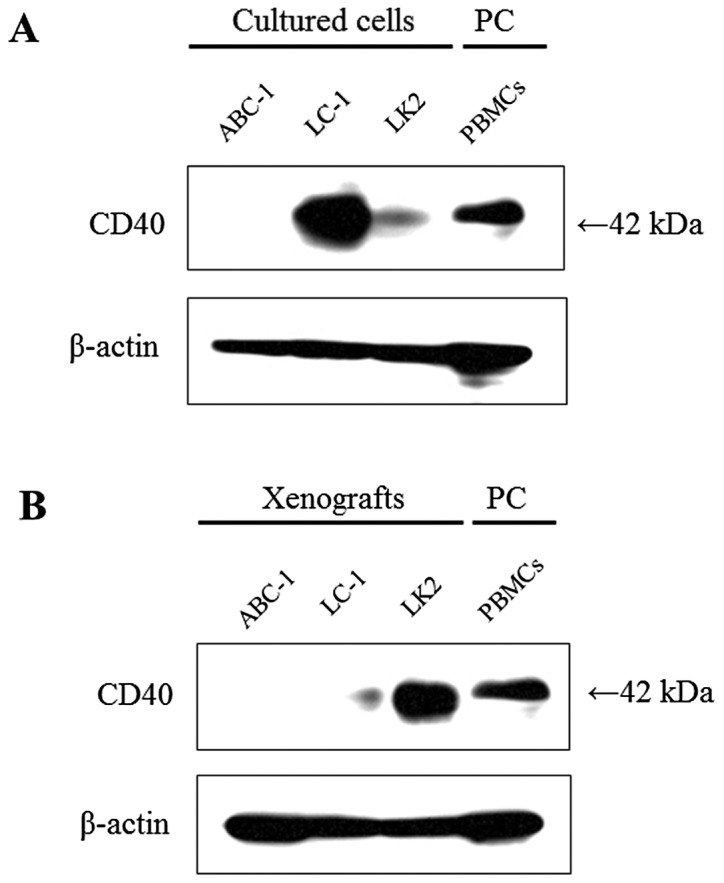
Western blotting for CD40 validation. (A) Lysates from human lung cancer cell lines were subjected to western blotting. Lysates from PBMCs were used as a positive control for CD40. CD40 was strongly detected at 42 kDa in lysates from LC-1, LK2 and in PBMCs. (B) Western blotting for CD40 in lysates from CB17/SCID mouse xenograft models. Lysates from implanted tumors derived from human lung cancer cell lines were subjected to western blotting. Each lung cell line was implanted subcutaneously into the left flanks of CB17/SCID mice. PBMCs, peripheral blood mononuclear cells.

CD40 expression was strongly detected at 42 kDa in cultured LC-1 cells; it was moderately detected in cultured LK2 cells, but it was not detected in ABC-1 cells (Fig. 5A). When compared with the results from cultured cell lines, CD40 expression was markedly downregulated in the LC-1 xenograft, upregulated in the LK2 xenograft, but it was not detected in the ABC-1 xenograft (Fig. 5B).

**Figure 6:**
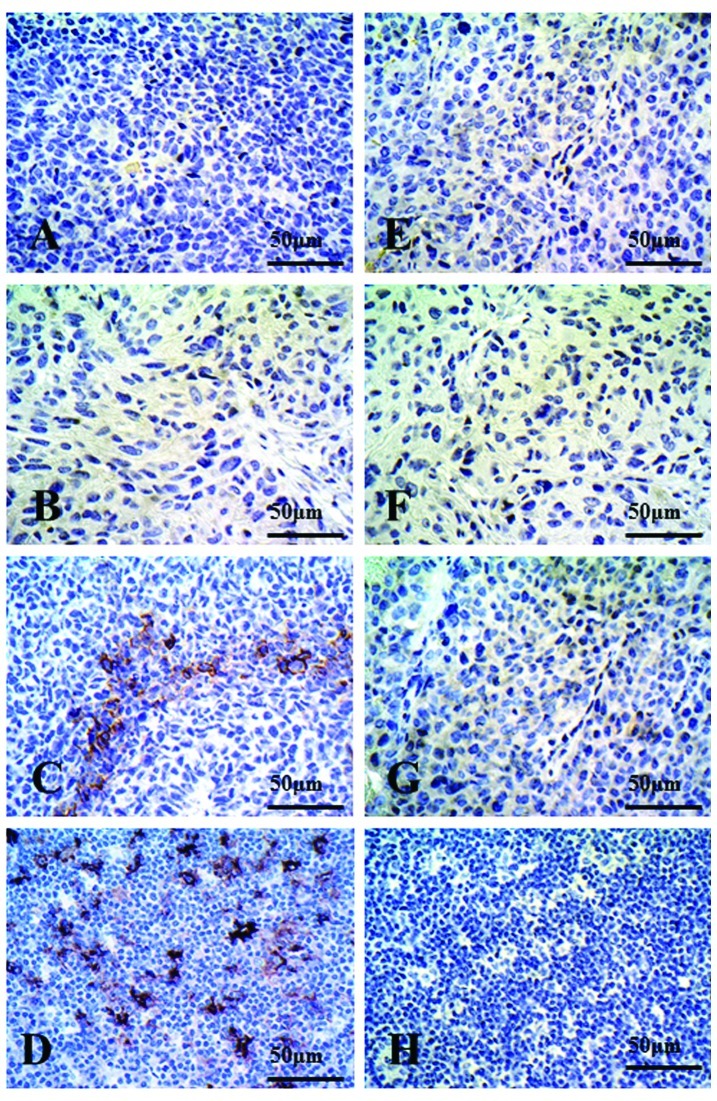
Immunohistochemical staining for CD40 in SCID xenograft models and human lymph nodes. (A) ABC-1 xenograft not expressing CD40, with anti-CD40 mouse monoclonal antibody used as primary antibody. (B) LC-1 xenograft not expressing CD40, with anti-CD40 mouse monoclonal antibody used as primary antibody. (C) LK2 xenograft expressing CD40, with anti-CD40 mouse monoclonal antibody used as primary antibody. (D) Human lymph nodes, with anti-CD40 mouse monoclonal antibody used as primary antibody. (E) ABC-1 xenograft not expressing CD40, with mouse isotype-matched negative control IgG used as primary antibody. (F) LC-1 xenograft not expressing CD40, with mouse isotype-matched negative control IgG used as primary antibody. (G) LK2 xenograft not expressing CD40, with mouse isotype-matched negative control IgG used as primary antibody. (H) Human lymph nodes, with mouse isotype-matched negative control IgG used as primary antibody.

CD40 immunostaining was not detected in the ABC-1 xenograft (Fig. 6A) or in the LC-1 xenograft (Fig. 6B). On the other hand, CD40 staining was strongly detected in the LK2 xenograft with heterogeneity (Fig. 6C) and in human lymph nodes (Fig. 6D). There were no CD40-positive cells in any of the xenografts or in human lymph nodes when control IgG was used as a primary antibody (Fig. 6E-H).

## Discussion

Immunohistochemical staining is associated with several problems due to the sensitivity of the technical process. In particular, results cannot be unified when there is no internal positive control. We have repeatedly performed immunohistochemistry and have often found the results to be inconsistent with *in vitro* analysis data. Our aim is to unify the results from any laboratory and to ensure that immunohistochemistry is objective.

CD154 (CD40 ligand) is known to be expressed in normal lymphocytes (3–5), while recent reports have found that CD154 is expressed in various cancer cells (3,16,17). We initially attempted immunostaining for CD154 in normal human tonsils and lymph nodes, using C-20 (18) and TRAP1:IM1842 (17), respectively, as primary antibodies, but no CD154 staining was seen (data not shown). Thus, we attempted to establish a new positive control tissue section for CD154, and we assessed the efficiency of these two primary antibodies for CD154 recognition.

Using western blot analysis, rhsCD154 was used as a positive control for CD154. CD154 was strongly detected at 36 kDa as a homotrimer and at 16.3 kDa as a monomer (21). We expected CD154 expression to be detected in PBMCs, as CD154 is expressed in normal lymphocytes (3–5), but with western blot analysis, CD154 expression was not detected in PBMCs.

It has been reported that 0.2% of normal human peripheral blood CD4-positive T lymphocytes are positive for CD154 (22,23). This suggests that CD154 is scarcely detected at the protein level in lysates from PBMCs. C-20:sc978 is able to detect rhsCD154 protein. These results suggest that normal human tonsils or lymph nodes are not suitable as positive controls for CD154 immunohistochemical staining. On the other hand, when TRAP1:IM1842 was used as a primary antibody, there was no band detected for rhsCD154 (data not shown). However, TRAP1:IM1842 is only recommended for flow cytometric analysis, and is thus not suitable for western blotting.

The PC10 xenograft immunostaining data indicated that C-20:sc-978 was neutralized by rhsCD154 protein, and the affinity between rhsCD154 and C-20:sc-978 is quite high (Fig. 2B and C). It is certain that the PC10 xenograft expresses CD154, while the LK2 xenograft immunostaining data suggests that it could be used as the CD154-negative sample. These findings suggest that the results of immunostaining are consistent with the results of western blot analysis using C-20:sc978 as a primary antibody.

These results (PC10 is CD154-positive and LK2 is CD154-negative) should be preserved with other primary antibodies, such as TRAP1:IM1842, in immunohistochemical staining for CD154. In the present study, however, TRAP1:IM1842 was not suitable for western blot analysis, as the PC10 xenograft exhibited no staining. When TRAP1:IM1842 was used, the results of immunostaining for CD154 were consistent with those using C-20:sc978, but the staining level was very weak in xenograft PC10 (Fig. 2F) when compared with the C-20:sc978 results (Fig. 2B). Although the action of TRAP1:IM1842 is known for flow cytometric analysis, it is not suitable for western blot analysis or immunohistochemical staining.

The present observations suggest the following: i) C-20:sc-978 is suitable for use as a primary antibody; ii) PC10 SCID xenografts may be used as a positive control tissue specimen for CD154 immunostaining; and iii) LK2 SCID xenografts may be used as a negative control tissue specimen for CD154 immunostaining. These measures allow for simultaneous evaluation of reagent controls, including primary antibodies, and tissue controls, such as xenografts. Based on these findings, western blot analysis was most suitable to confirm the target protein (CD154), and gave results consistent with those of immunohistochemistry. The advantage of this method is that the same antibody is used as the primary antibody in both techniques, although different primary antibodies could be used. In such a case, however, an alternative experimental method should be used to confirm target gene expression, such as RT-PCR or flow cytometric analysis.

The differences in gene expression between cultured cells *in vitro* and implanted cells were noteworthy. Although the expression of CD154 in NSCLC cell lines did not vary, confirmation of gene expression by western blot analysis both *in vitro* and *in vivo* is critical.

We then determined whether these methods could be applied to other target proteins, such as CD40. Markedly, CD40 expression in the lysates from SCID xenografts differed from that in cultured cell lines. These phenomena suggest that cancer cell implantation to the SCID mouse itself alters the gene profile. Previous reports have shown a clear difference in methylation pattern between cultured cancer cell lines and nude mice xenografts (24). Variations in methylation pattern were paralleled by variations in gene expression between cancer cell lines and mouse xenografts (24). Expression of CD40 may also depend on cytokines (25).

CD40 immunostaining was consistent with the results of xenograft CD40 expression on western blot analysis. This suggests that western blot analyses may exhibit different results from immunostaining with cultured cell lines and xenografts, and thus western blotting using lysates from xenografts should be performed to assess consistency with the immunostaining results using SCID xenografts. We established SCID xenografts and prepared xenograft blocks as formalin-fixed, paraffin-embedded tissue, and immunostaining may be performed using paraffin-embedded tissue, cell blocks or acetone-fixed live cells. The most marked difference between xenografts and cell lines was that the xenografts have the morphological characteristics of a tumor. For accuracy, positive or negative tissue controls should be prepared in the same manner as patient samples.

We used anti-CD40 mouse monoclonal antibody (11E9) for CD40 immunostaining in SCID xenografts and human lymph nodes. The results suggested that mouse monoclonal antibody may be used as a primary antibody against SCID xenograft samples without background staining due to endogenous immunoglobulin. To our knowledge, there have been few reports on the use of mouse monoclonal antibodies as primary antibodies in immunohistochemistry against SCID xenografts. The advantage of this method is that SCID xenografts can be obtained using a simple procedure.

In conclusion, we propose a control concept for immunohistochemistry based on western blot analysis, by investigating CD154 and CD40 expression in NSCLC using SCID xenograft models. The present results demonstrate that this method is objectively suitable.
